# Anti-Double-Stranded DNA IgG Participates in Renal Fibrosis through Suppressing the Suppressor of Cytokine Signaling 1 Signals

**DOI:** 10.3389/fimmu.2017.00610

**Published:** 2017-05-31

**Authors:** Ping Wang, Jie Yang, Fang Tong, Zhaoyang Duan, Xingyin Liu, Linlin Xia, Ke Li, Yumin Xia

**Affiliations:** ^1^Core Research Laboratory, The Second Affiliated Hospital, School of Medicine, Xi’an Jiaotong University, Xi’an, China; ^2^Department of Nephrology, Tangdu Hospital, Fourth Military Medical University, Xi’an, China; ^3^Department of Immunology and Microbiology, Wannan Medical College, Wuhu, China; ^4^Department of Nephrology, The Second Affiliated Hospital, School of Medicine, Xi’an Jiaotong University, Xi’an, China; ^5^Department of Pathogenic Biology, Nanjing Medical University, Nanjing, China; ^6^Department of Dermatology, The Second Affiliated Hospital, School of Medicine, Xi’an Jiaotong University, Xi’an, China

**Keywords:** anti-double-stranded DNA IgG, suppressor of cytokine signaling 1, Janus kinase 2, signal transducer and activator of transcription 1, peptide, lupus nephritis, renal fibrosis

## Abstract

Suppressor of cytokine signaling 1 (SOCS1) participates in renal fibrosis by downregulating Janus kinase 2 (JAK2)/signal transducer and activator of transcription 1 (STAT1)-mediated cytokine signaling. Recently, it was found that anti-double-stranded DNA (dsDNA) IgG induces the synthesis of profibrotic cytokines by renal cells. To explore the potential effect of anti-dsDNA IgG on SOCS1-mediated renal fibrosis, kidney tissues were collected from patients with lupus nephritis (LN) as well as MRL/lpr lupus-prone mice. The SOCS1 expression was evaluated in tissue samples. In addition, SCID mice were injected with anti-dsDNA IgG, followed by evaluation of SOCS1 levels. Renal resident cells were cultured *in vitro*, receiving the stimulation of anti-dsDNA IgG and then the measurement of SOCS1, JAK2, STAT1α, and profibrotic cytokines. Moreover, the binding of anti-dsDNA IgG to SOCS1 kinase inhibitory region (KIR) peptide was analyzed by surface plasmon resonance. We found that SOCS1 expression was inhibited, but JAK2/STAT1 activation was prominent in the kidney tissues of patients with LN, MRL/lpr mice, or anti-dsDNA IgG-injected SCID mice. The cultured renal cells also showed SOCS1 downregulation, JAK2/STAT1 activation, and profibrotic cytokine promotion upon anti-dsDNA IgG stimulation. Surprisingly, anti-dsDNA IgG showed high affinity to KIR peptide and competed with JAK2 loop for KIR. Additionally, a DNA-mimicking peptide (ALW) blocked the binding of anti-dsDNA IgG to KIR, and even partially abrogated the activation of JAK2/STAT1α signals and the expression of profibrotic cytokines in SCID mice. In conclusion, anti-dsDNA IgG downregulates SOCS1 expression, activates JAK2/STAT1 signals, and contributes to renal fibrosis; its peptide blockade may restore the SOCS1 inhibitory effect on the production of profibrotic cytokine, and finally ameliorate renal fibrosis in LN.

## Introduction

As the most common internal complication in patients with systemic lupus erythematosus (SLE) (1), lupus nephritis (LN) is essentially a chronic inflammation in kidneys. The histological changes of LN are characterized by a spectrum of morphologic abnormalities in both glomerular and tubulointerstitial regions. Although LN is classified into Class I to Class VI according to pathological patterns (2), renal fibrosis is definitely the common final outcome at the end stage (1). The fibrotic lesions are associated strongly with poor outcome of patients with LN (3). During the progression of renal fibrosis, profibrotic cytokines are continuously released, and consequently enhance the phenotype changes of resident cells as well as the accumulation of extracellular matrix (3, 4). Therefore, elucidation of the pathogenesis of renal fibrosis, especially the regulation of profibrotic cytokines, is important in the development of therapeutic strategies for patients with LN.

Many studies demonstrated that anti-double stranded DNA (dsDNA) antibodies correlate closely with LN (5–8). Recent classification criteria for SLE were established by the Systemic Lupus International Collaborating Clinics, accepting biopsy-proven LN in combination with the presence of anti-dsDNA antibodies in sera as an independent criterion (9). In fact, anti-dsDNA IgG can bind to cell surface or intracellular molecules, penetrate living kidney cells and modulate gene expression, and even enhance renal cell proliferation (6–8). Moreover, anti-dsDNA IgG contributes to renal fibrosis by inducing a myofibroblast-like phenotype of mesangial cells and also enhancing the synthesis of proinflammatory cytokines and fibrotic factors in renal cells (10–13). Anti-dsDNA antibodies even induce the secretion of interleukin (IL)-1β, IL-6, and tumor necrosis factor-α in mesangial and endothelial cells, which further promotes the expression of downstream cytokines and fibrotic molecules such as hyaluronan and adhesion proteins (7). Therefore, anti-dsDNA IgG can interact with intracellular molecules and directly affect renal inflammatory and fibrotic progresses in LN.

Suppressor of cytokine signaling 1 (SOCS1) suppresses the Janus kinase 2 (JAK2) pathway, thereby downregulating signal transducer and activator of transcription 1 (STAT1) (14). Inhibition of SOCS1 leads to over-activation of the JAK2 pathway (15). The JAK and downstream STAT1 molecules participate in the proliferation and cytokine production of various types of cells. Upon activation, JAK2 phosphorylates tyrosine residues on the receptors (e.g., interferon receptors) and creates binding sites for SH2 domains (15). The SH2-containing STAT1 is subsequently activated by JAK2 and next induces transcription of target genes including those that code profibrotic cytokines (16). The kinase inhibitory region (KIR) of SOCS1 can bind to the loop region of JAK2 and then suppress the JAK catalytic activity, thereby resulting in the inhibition of JAK2 phosphorylation and STAT1 activation (17).

Although it was occasionally reported that the SOCS1 mRNA level increases in peripheral blood mononuclear cells of patients with SLE (18, 19), many more studies revealed significant downregulation of SOCS1 expression in these cells (20–22), and steroid administration to these cells upregulates SOCS1 expression in a dose- and time-dependent manner (22). Moreover, in different murine models of SLE, SOCS1 suppression contributes to the progression of lupus disease (23–25). Currently, SOCS1 has been proven to be an inhibitor of renal fibrosis. In LN, renal SOCS1 expression alters negatively with the levels of fibrotic cytokines including transforming growth factor (TGF)-β (26). The overexpression of SOCS1 inhibits tubular epithelial cell-myofibroblast transdifferentiation by decreasing the expression of IL-1β and oncostatin M (14). Even more, SOCS1 expression decreases significantly, whereas the expression of profibrotic genes increases in kidney biopsies from patients with LN (27). miR-150 can promote renal fibrosis in LN by inhibiting both mRNA and protein levels of SOCS1 (27). These results strongly suggested that SOCS1 plays an important role in the pathogenesis of SLE as well as renal fibrosis of LN.

The JAK2/STAT1 pathway mediates the renal damage in lupus models induced by anti-dsDNA antibodies. In MRL/lpr mice, the selective JAK2 inhibitor tyrphostin AG490 significantly inhibited the phosphorylation of JAK2 and STAT1, accompanied by a decrease in proteinuria, renal histological lesions, and serum titer as well as glomerular deposition of anti-dsDNA IgG (28). Moreover, STAT1 deficiency in the lupus-like chronic graft-versus-host disease model led to a prolonged but amplified increase of anti-dsDNA IgG in sera and also higher proteinuria and mortality (29). This phenomenon might be due to the imbalance of STAT1 and other STATs after downstream inhibition of the JAK2/STAT1 pathway (29). However, these results strongly suggested that the JAK2/STAT1 pathway regulates the pathogenic role of anti-dsDNA antibodies in LN. Actually, by using BALB/c mice intravenously injected with anti-dsDNA IgG, the cross-reaction of anti-dsDNA antibodies with mesangial matrix or glomerular basement membrane is proven critical for initiating glomerular inflammation (30). Additionally, SOCS1 has been found to mediate double-strand break repair of DNA and to preserve the genomic stability (31). Hence, these findings highlight another potential protective role of SOCS1 in LN, which may be related to the nephritogenicity of anti-dsDNA antibodies.

Considering the facts that anti-dsDNA IgG can penetrate living cells and interact with intracellular molecules (6–8, 10–13), we speculate that anti-dsDNA IgG may also modulate SOCS1 signals. The purpose of this study was to investigate the potential effect of anti-dsDNA IgG on the regulation of SOCS1 signals and also the molecular mechanism underlying SOCS1-KIR and JAK2/STAT1 interaction in the pathogenesis of renal fibrosis.

## Materials and Methods

### Tissue and Serum Samples

Renal biopsies were obtained from patients with LN (*n* = 7), who had serum positivity of anti-dsDNA IgG (Table S1 in Supplementary Material). The diagnosis of LN was made based on the histological features of renal biopsies. For normal human controls, sera and non-lesional tissues were taken from patients (*n* = 5) who underwent surgical nephrectomy due to renal cell carcinoma or renal rupture (Table S1 in Supplementary Material). There was no statistical difference in term of age between the LN patients and the controls (*p* > 0.05). These control samples showed no evident inflammatory or fibrotic appearance under the microscope. The MRL/lpr and MRL/MpJ mice (female, 12 or 24 weeks old, *n* = 5 each group) as well as SCID mice (female, 8 weeks old, *n* = 5 each group) were bred in the animal facility of Xi’an Jiaotong University Health Science Center. Urine, sera, and renal tissues were harvested from MRL/lpr and MRL/MpJ mice. Proteinuria was quantitated by the Coomassie brilliant blue method (32). All data about MRL/lpr and MRL/MpJ mice referred to that at age of 24 weeks unless otherwise noted. These SCID mice received intravenous injections of anti-dsDNA or control IgG at a dose of 100 µg twice weekly, which lasted for 4 weeks (*n* = 5 each group). Then, both serum and kidney samples were collected from SCID mice. This study was carried out in accordance with the recommendations of the guidelines of the Hospital Research Ethics Committee with written informed consent from all subjects. All subjects gave written informed consent in accordance with the Declaration of Helsinki. The protocol was approved by the Hospital Research Ethics Committee.

### Immunohistochemistry (IHC) and Immunofluorescence (IF)

As described previously (8), IHC was performed for renal IgG deposition. For detection of SOCS1, paraffin sections were incubated with rabbit anti-mouse SOCS1 (2 µg/ml; Abcam, Cambridge, MA, USA), and then with polymer-horseradish peroxidase conjugated goat anti-rabbit IgG (1 µg/ml; DAKO, Glostrup, Denmark). A brown color was developed with 3,30-diaminobenzidine chromogen substrate (DAKO).

Immunofluorescence was carried out for both IgG deposition and SOCS1 expression in frozen sections. Routinely processed sections were incubated with rabbit anti-mouse SOCS1 and then fluorescein isothiocyanate (FITC)-conjugated goat anti-rabbit IgG (2 µg/ml; Southern Biotech, Birmingham, AL, USA) in order, or directly with goat anti-mouse IgG-FITC (Southern Biotech) alone. Sections were viewed under a digital fluorescent microscope (Carl Zeiss, Jena, Germany).

### Cell Culture and Anti-dsDNA IgG Stimulation

Immortal murine mesangial cells and glomerular endothelial cells (Jieqing Biotech, Wuhan, China) were cultured in RPMI 1640 medium supplemented with 10% fetal bovine serum (8, 33). Immortal murine proximal tubular epithelial cells (kindly provided by Dr. Cheng Pan, Wuhan University, China) were cultured in Dulbecco’s modified Eagle’s medium as described previously (34).

By using a HiTrap purification kit (GE Healthcare, Port Washington, NY, USA) (8), anti-dsDNA IgG was purified from the culture supernatants of murine WJ31 hydridoma clone (IgG2a; Jieqing Biotech). Control murine IgG (IgG2a; Jieqing Biotech) was verified by enzyme-linked immunosorbent assay (ELISA) to determine that there was no binding to DNA antigen (data not shown). Cells were starved routinely before the 2-day stimulation of anti-dsDNA IgG or control IgG (2 µg/ml). In some experiments, mesangial cells were stimulated with anti-dsDNA IgG (2 µg/ml) that was premixed with d-form (ALWPPNLHAWVP) or scrambled (PLPHNPWVLAAW) ALW peptide (1 µg/ml). The cell viability of three types of cells was measured using PrestoBlue viability reagent (Life Technologies, Carlsbad, CA, USA).

### Real-time PCR

As previously described (8), total RNA was extracted from fresh tissues or cultured cells, followed by reverse transcription. The 7900HT Fast PCR System (Applied Biosystems, Carlsbad, CA, USA) was used for PCR, and SYBR Green Master Mixes (Invitrogen, Grand Island, NY, USA) was used as fluorescent dye. The sequences of primers (Jieqing Biotech) are listed in Table S2 in Supplementary Material.

### Western Blotting and Immunoprecipitation

Fresh tissues and cell cultures were extracted for lysates with the addition of protease inhibitor cocktail (Thermo Scientific, Waltham, MA, USA). Lysate samples were run in gel at denatured and reduced condition and then transferred to a PVDF membrane (EMD Millipore, Billerica, MA, USA). The rabbit primary antibodies (2 µg/ml; Abcam) and horseradish peroxidase-goat anti-rabbit IgG (2 µg/ml; Southern Biotech) were applied in order. The band intensities were quantitated by ImageJ 1.61u software (National Institutes of Health, Bethesda, MD, USA) and normalized to β-actin values accordingly. The intensities of blank groups were set as 1 (baseline), and the relative intensities were calculated in other groups.

By using a protein G kit (Roche, Indianapolis, IN, USA), immunoprecipitation was performed with the lysates of mesangial cells. Briefly, lysates were mixed with anti-DNA IgG (or control) and then incubated at 4°C overnight. Protein G suspension was added to the mixtures. The supernatants were discarded after centrifuging while the pellets were re-suspended, followed by Western blotting.

### Enzyme-Linked Immunosorbent Assays

Common ELISAs (for binding to dsDNA or peptide) and inhibition ELISAs were performed as previously described (8, 35). Briefly, the KIR ELISA was carried out by adding biotinylated KIR peptide (DTHFRTFRSHSDYRRI, 5 µg/ml) to streptavidin-coated 96-well plates. For inhibition with KIR (or ALW) peptide, anti-dsDNA IgG (2 µg/ml) was pre-incubated with serially diluted peptide (from 0.04 to 80 µg/ml), and then transferred to antigen-coated 96-well plates. Alkaline phosphatase-conjugated goat anti-mouse IgG (2 µg/ml; Southern Biotech) was used as secondary antibody. Similarly, for inhibition with anti-dsDNA IgG, the FITC-labeled JAK2 loop peptide (LPQDKEYYKVKEP, 0.2 µg/ml) was pre-incubated with serially diluted anti-dsDNA IgG (from 0.02 to 20 µg/ml) before transfer to KIR-coated plates, which were detected for fluorescein intensity under a Perkin Elmer Reader (Winpact Scientific, Irvine, CA, USA). Cell surface ELISA was performed by growing mesangial cells in 96-well plates (8, 35). All peptides were synthesized by Sangon Biotech Company (Shanghai, China).

### Surface Plasmon Resonance

The Biacore 3000 instrument (Biacore, Piscataway, NJ, USA) was used for the quantitation of binding affinities (8, 35). For anti-dsDNA IgG binding to KIR peptide, anti-dsDNA IgG (10 nM in MES buffer) was immobilized on a CM sensor chip (GE Healthcare, Port Washington, NY, USA), and then KIR peptide (0–250 nM in HEPES buffer) was run on chip. For JAK2 loop peptide binding to KIR peptide, the biotinylated KIR peptide (5 nM) was immobilized on streptavidin-coated sensor chip, followed by injection of JAK2 loop peptide (0–250 nM). A simple Langmuir model (A + B ↔ AB) was used for calculating binding kinetics.

### Matrix-Assisted Laser Desorption/Ionization (MALDI)–Time of Flight Mass Spectrometry

Matrix-assisted laser desorption/ionization–time of flight mass spectrometry was performed for identifying the sequences of peptide fragments. As described previously (36), an ABI 4800 Analyzer (AB Science, Foster City, CA, USA) was used for such analysis. The ALW (l-form or d-form) and KIR peptides (2 µg/ml) were mixed with anti-dsDNA IgG or control IgG (molar ratio = 1:5), respectively. The mixtures were incubated at 37°C for 30 min. Then, these samples were diluted in distilled water (1:100) and then mixed with the α-cyano-4-hydroxycinnamic acid matrix at a ratio of 1:1. Samples in triplicate were then loaded to the MALDI target. Mass spectrum was produced on 80 individual spectra of each spot.

### Statistical Methods

Data were expressed as mean ± SEM. Statistical analysis was performed using the STATA 10.0 software (StataCorp, College Station, TX, USA). Unpaired and two-tailed *t* test was used for comparing the differences between two groups after significant differences were found in an ANOVA. The differences were considered significant at *p* < 0.05.

## Results

### SOCS1 Expression Decreases in the Glomeruli with Anti-dsDNA IgG Deposition

**Figure 1:**
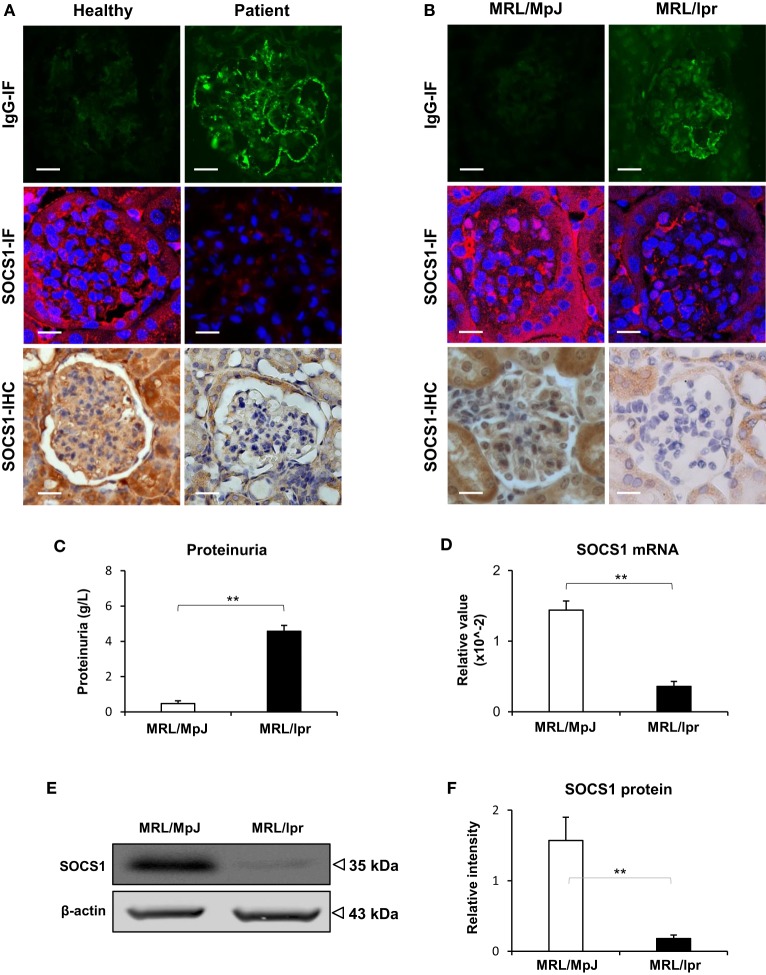
**Renal suppressor of cytokine signaling 1 (SOCS1) expression decreases in patients with lupus nephritis (LN) and MRL/lpr mice**. The renal IgG deposition and SOCS1 expression levels were detected in normal human controls (*n* = 5), patients with LN (*n* = 7), MRL/MpJ (*n* = 5) mice, and MRL/lpr (*n* = 5) mice. **(A)** By immunohistochemistry (IHC) and immunofluorescence (IF), IgG deposition and SOCS1 expression were detected in the frozen sections of human kidneys. **(B)** Similarly, IgG and SOCS1 were detected in kidney samples from mice. **(C)** Proteinuria was determined in these mice. **(D)** The SOCS1 mRNA levels were determined in fresh kidney tissues of mice. **(E,F)** SOCS1 protein was analyzed in kidney tissues of mice, followed by ImageJ quantitation of Western blot bands. Data points and error bars represent mean ± SEM. Representative images are shown. Scale bar = 5 µm; **p < 0.01.

The LN patients and MRL/lpr mice (at age of 24 weeks) recruited into this study had high serum titers of anti-dsDNA IgG (Figure S1 in Supplementary Material). By IF, a strong positivity in the glomeruli of LN patients or MRL/lpr mice was noted, while normal human donors or MRL/MpJ mice exhibited slight or non-specific staining (Figures 1A,B). Both IHC and IF were then performed with kidney samples, showing less glomerular expression of SOCS1 in LN patients and MRL/lpr mice when compared with their controls accordingly (Figures 1A,B). The MRL/lpr mice had higher proteinuria than that of MRL/MpJ mice (Figure 1C). Using real-time PCR, the mRNA levels of SOCS1 were quantitated with fresh kidney tissues from these two mouse strains, displaying lower renal levels in MRL/lpr mice (Figure 1D). Similarly, the protein levels of SOCS1 were also lower in MRL/lpr mice by Western blotting (Figures 1E,F). The MRL/lpr and MRL/MpJ mice at age of 12 weeks were also assessed, showing comparable levels of both proteinuria and renal SOCS1 protein between the two groups (Figure S2 in Supplementary Material).

**Figure 2:**
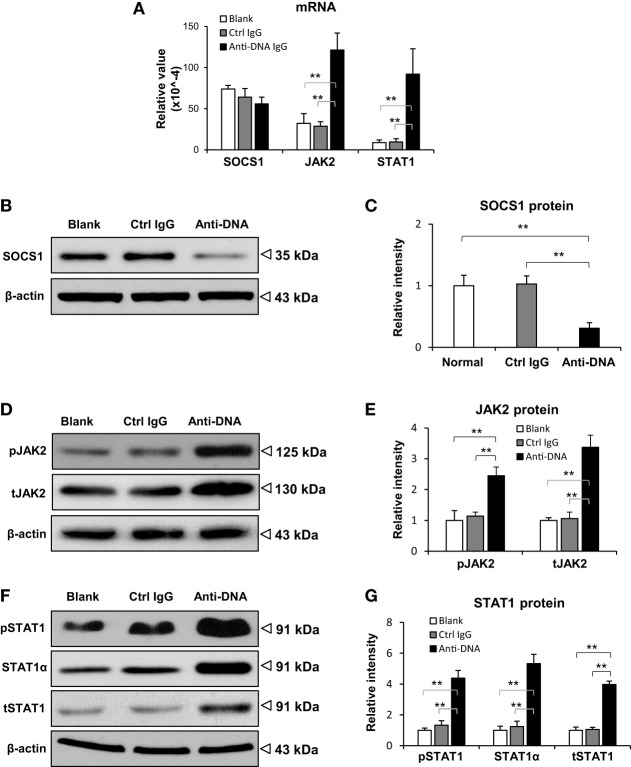
**Evaluation of suppressor of cytokine signaling 1 (SOCS1) and Janus kinase 2 (JAK2)/signal transducer and activator of transcription 1 (STAT1) expression levels in kidneys of SCID mice**. Mice were intravenously injected with control or anti-dsDNA IgG before tissular analysis. **(A)** The mRNA levels of SOCS1, JAK2, and STAT1 were measured in fresh kidney tissues. By Western blotting, the proteins of SOCS1 **(B,C)**, JAK2 **(D,E)**, and STAT1 **(F,G)** were determined in kidney tissues, followed by ImageJ quantitation of band intensities. There were no differences between the mice in blank and control IgG groups (*p* > 0.05). There were five mice in each group. Data points and error bars represent mean ± SEM. Representative images are shown (**p < 0.01).

To exclude the potential effect of other lupus-related autoantibodies on SOCS1 expression, SCID mice were intravenously injected with murine anti-dsDNA IgG. These mice exhibited IgG deposition in glomeruli while it was negative in the control IgG-injected mice (Figure S3 in Supplementary Material). The expression of SOCS1 as well as downstream JAK2 and STAT1 were assessed in their kidneys. We found that the mRNA levels of SOCS1 were comparable among the three groups, but the mRNA levels of JAK2 and STAT1 were enhanced upon anti-dsDNA IgG injection (Figure 2A). Moreover, the protein levels of SOCS1 decreased significantly, while the protein levels of JAK2 (phosphorylated and total forms) and STAT1 (α-, phosphorylated, and total forms) were higher in the anti-dsDNA IgG group (Figures 2B–G).

**Figure 3:**
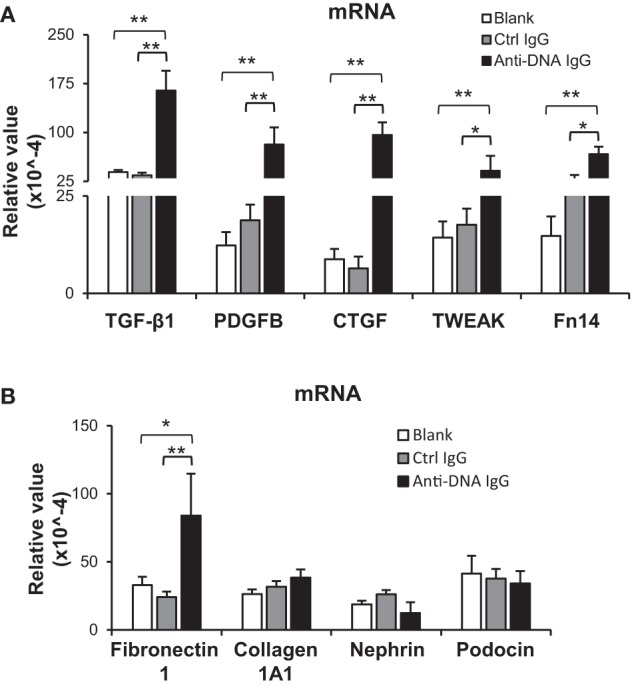
**Evaluation of renal mRNA levels of profibrotic factors in SCID mice**. Mice were intravenously injected with control or anti-dsDNA IgG before tissue collection. **(A)** The mRNA expression levels of profibrotic cytokines [transforming growth factor (TGF)-β1, platelet-derived growth factor subunit B (PDGFB), and connective tissue growth factor (CTGF)] and proinflammatory factors (TWEAK and Fn14) were determined in fresh kidney tissues. **(B)** The mRNA levels of extracellular matrix components (fibronectin 1 and collagen 1A1) as well as structural markers (nephrin and podocin) were determined similarly. There were no differences between the mice in blank and control IgG groups (*p* > 0.05). There were five mice in each group. Data points and error bars represent mean ± SEM (*p < 0.05, **p < 0.01).

Furthermore, the profibrotic factors including TGF-β1, platelet-derived growth factor subunit B (PDGFB), and connective tissue growth factor (CTGF) were determined in the kidneys of SCID mice. It showed that their mRNA levels were elevated in anti-dsDNA IgG-injected mice when compared with the two controls (Figure 3A). The mRNA of proinflammatory cytokine TWEAK (tumor necrosis factor-related weak inducer of apoptosis) and its receptor Fn14 (fibroblast growth factor-inducible 14) was also analyzed, verifying more mRNA expression in the anti-dsDNA IgG group (Figure 3A). These mice were even analyzed for the mRNA levels of extracellular matrix components (fibronectin 1, collagen 1A1) and structural markers of podocytes (nephrin, podocin). It was observed that only fibronectin 1 mRNA was promoted upon anti-dsDNA IgG injection (Figure 3B). The effect of anti-dsDNA IgG on proinflammatory factors was also mirrored by *in vitro* experiments showing that anti-dsDNA IgG induced higher mRNA expression of PDGFB, CTGF, and Fn14 in murine mesangial cells (Figure S4 in Supplementary Material).

### Anti-dsDNA IgG Blocks the SOCS1 Signals in Kidney Cells

**Figure 4:**
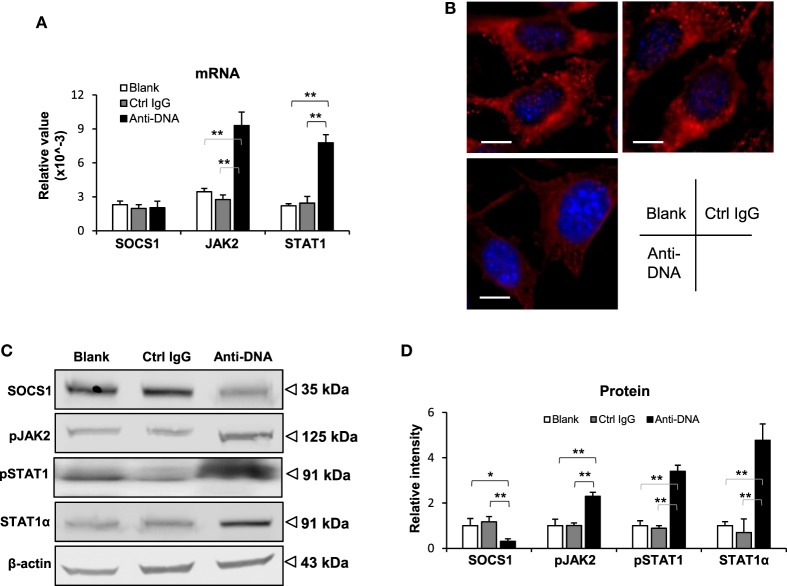
**Evaluation of suppressor of cytokine signaling 1 (SOCS1) and Janus kinase 2 (JAK2)/signal transducer and activator of transcription 1 (STAT1) expression levels in mesangial cells**. Cells were cultured *in vitro* and stimulated by control or anti-dsDNA IgG (2 µg/ml, 2 days). **(A)** The mRNA levels of SOCS1, JAK2, and STAT1 were determined in these cells. **(B)** By confocal microscopy, SOCS1 expression was detected in cells. **(C)** Western blotting was performed to detect the proteins of SOCS1, JAK2, and STAT1 in cell lysates. **(D)** The intensities of the Western blot bands were quantitated by ImageJ software. Data were from three independent experiments. Data points and error bars represent mean ± SEM. Representative images are shown. Scale bar = 5 µm (*p < 0.05, **p < 0.01).

To further determine the effect of anti-dsDNA IgG on SOCS1 signals, murine kidney cells were cultured *in vitro*. After anti-dsDNA IgG stimulation, mesangial cells showed SOCS1 mRNA level similar to that in other groups, but higher mRNA levels of JAK2 and STAT1 when compared with other groups (Figure 4A). By IF, mesangial cells expressed less SOCS1 upon anti-dsDNA IgG stimulation (Figure 4B). The proteins of SOCS1, phospho-JAK2, phospho-STAT1, and STAT1α were also determined, showing a decrease in SOCS1 but an increase in phospho-JAK2, phospho-STAT1, and STAT1α in the anti-dsDNA IgG group (Figures 4C,D). Similar results were seen with glomerular endothelial cells (Figure S5 in Supplementary Material) and proximal tubular epithelial cells (Figure S6 in Supplementary Material). Cell viability was determined in these three types of cells, showing no changes upon anti-dsDNA IgG or d-form ALW stimulation (Figure S7 in Supplementary Material).

### Anti-dsDNA IgG Competes with JAK2 Loop for SOCS1-KIR

**Figure 5:**
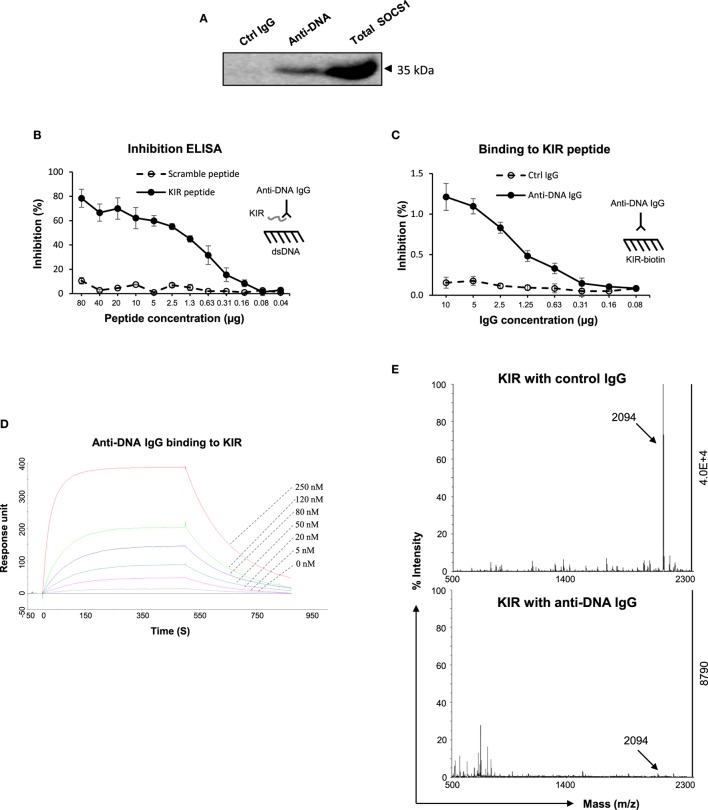
**The interactions between anti-dsDNA IgG and suppressor of cytokine signaling 1 (SOCS1) [or kinase inhibitory region (KIR) peptide]**. **(A)** By immunoprecipitation and Western blotting, the lysate proteins in mesangial cells were selected by anti-DNA IgG and then detected with anti-SOCS1 antibody. **(B)** By inhibition enzyme-linked immunosorbent assay (ELISA), the binding of anti-dsDNA IgG to double-stranded DNA (dsDNA) antigen was measured upon addition of KIR (or scrambled) peptide. **(C)** The binding of anti-dsDNA IgG to biotinilyated KIR peptide was measured by direct ELISA. **(D)** Surface plasmon resonance was performed to quantitate the affinity of anti-dsDNA IgG to KIR peptide. Anti-dsDNA IgG was immobilized to sensor chip, followed by running KIR peptide (0–250 nM). Ka (1/Ms) = 3.65 × 10^4^, Kd (1/s) = 4 × 10^−3^, *K*_D_ (M) = 1.1 × 10^−7^, and Rmax (RU) = 351. **(E)** The catalytic effect of anti-dsDNA IgG on KIR peptide was detected by matrix-assisted laser desorption/ionization–time of flight mass spectrometry. Data were from three independent experiments. Data points and error bars represent mean ± SEM. Representative graphs are shown.

Since *in vitro* experiments indicated interference of anti-dsDNA IgG in the SOCS1 signals in kidney cells, we clarified the interaction between anti-dsDNA IgG and SOCS1 molecule. By immunoprecipitation and Western blotting, the lysate proteins in mesangial cells were selected by anti-DNA IgG and then detected with anti-SOCS1 antibody, showing a clear band at 35 kDa (Figure 5A). The KIR of SOCS1 was synthesized as a 16-mer peptide (2.09 kDa). Surprisingly, such KIR peptide efficiently inhibited binding of anti-dsDNA IgG to DNA antigen (Figure 5B). Moreover, anti-dsDNA IgG bound directly to biotinylated KIR peptide by ELISA (Figure 5C). Surface plasmon resonance was further performed to quantitate the binding affinity of anti-dsDNA IgG to KIR peptide, calculating binding kinetic of *K*_D_ (M) = 1.1 × 10^−7^ (Figure 5D). Moreover, KIR peptide was incubated with anti-dsDNA IgG or control IgG and then identified for fragment sequences by mass spectrometry. It showed that KIR peptide was almost catalyzed by anti-dsDNA IgG (Figure 5E).

**Figure 6:**
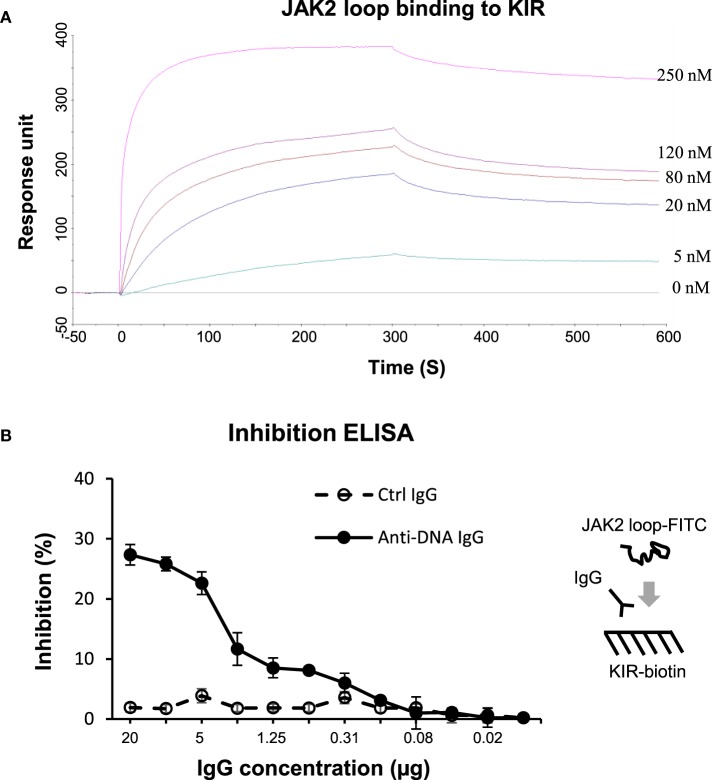
**The interactions between the suppressor of cytokine signaling 1 (SOCS1) kinase inhibitory region (KIR) and Janus kinase 2 (JAK2) loop peptides**. **(A)** Surface plasmon resonance was performed for the binding affinity of JAK2 loop peptide to biotinylated KIR peptide, which was immobilized on sensor chip. JAK2 loop peptide was run at a concentration of 0–250 nM. Ka (1/Ms) = 1.35 × 10^4^, Kd (1/s) = 6.03 × 10^−4^, *K*_D_ (M) = 4.46 × 10^−8^, and Rmax (RU) = 359. **(B)** By inhibition enzyme-linked immunosorbent assay (ELISA), the binding of fluorescein isothiocyanate (FITC)–JAK2 loop peptide to biotinylated KIR peptide was measured upon the addition of control or anti-dsDNA IgG. Data were from three independent experiments. Data points and error bars represent mean ± SEM. Representative graphs are shown.

The KIR peptide was further analyzed for specific affinity to JAK2 loop (13-mer, 1.64 kDa). By surface plasmon resonance, the JAK2 loop peptide bound to biotinylated KIR peptide with a *K*_D_ value of 4.46 × 10^−8^ (M) (Figure 6A). Additionally, inhibition ELISA was carried out by coating biotinylated KIR peptide to 96-well plates. The results showed that anti-dsDNA IgG inhibited the binding of FITC (0.39 kDa)–JAK2 loop to KIR peptide (Figure 6B). By cell surface ELISA, FITC-conjugated KIR peptide exhibited no binding to murine mesangial cells, glomerular endothelial cells or proximal tubular epithelial cells (Figure S8 in Supplementary Material).

### DNA-Mimicking Peptide Abrogates SOCS1 Downregulation Induced by Anti-dsDNA IgG

**Figure 7:**
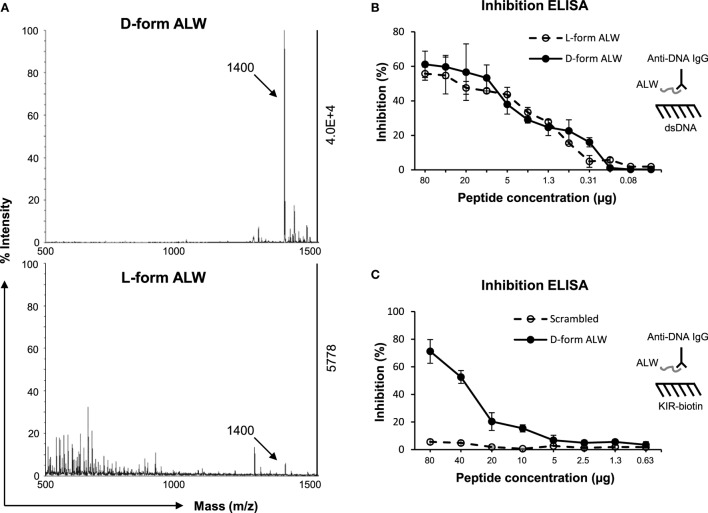
**The effect of d-form ALW peptide on anti-dsDNA IgG binding to double-stranded DNA (dsDNA) antigen or kinase inhibitory region (KIR) peptide**. **(A)** The catalytic effect of anti-dsDNA IgG on d-form or l-form ALW peptide was detected by matrix-assisted laser desorption/ionization-time of flight mass spectrometry. **(B)** Inhibition enzyme-linked immunosorbent assay (ELISA) was performed for the binding of anti-dsDNA IgG to dsDNA antigen upon the addition of ALW peptides. **(C)** Similarly, inhibition ELISA was performed for the binding of anti-dsDNA IgG to suppressor of cytokine signaling 1 (SOCS1)-KIR peptide upon the addition of d-form or scrambled ALW peptide. Data were from three independent experiments. Data points and error bars represent mean ± SEM. Representative graphs are shown.

Previously, a 12-mer peptide (abbreviated as “ALW”) was found to effectively block murine anti-dsDNA IgG binding to DNA antigen (35). However, ALW peptide also exhibits weakness as being susceptible to the catalyzing property of anti-dsDNA IgG (36). Therefore, ALW peptide was chemically modified by replacing two terminal residues of A (alanine) and P (proline) with d-form ones, respectively, which displayed enhanced resistance to catalysis (Figure 7A). Inhibition ELISA was performed similarly, showing that d-form ALW inherited the inhibitory property of the (natural) ALW as interfering anti-dsDNA IgG binding to DNA antigen (Figure 7B). Also, the d-form—but not the scrambled ALW—inhibited anti-dsDNA IgG binding to SOCS1-KIR peptide (Figure 7C), verifying the potential function of d-form ALW in blocking affinity of anti-dsDNA IgG to self-antigens that mimic DNA.

**Figure 8:**
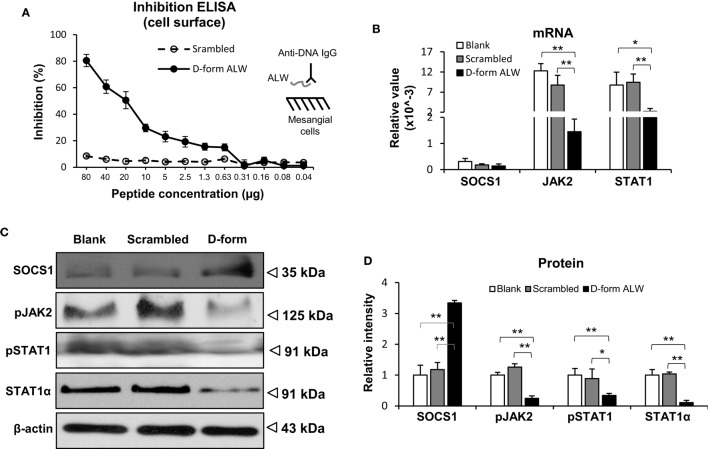
**The effect of d-form ALW peptide on anti-double-stranded DNA (dsDNA) IgG regulation of suppressor of cytokine signaling 1 (SOCS1) signals in mesangial cells**. Cells were cultured *in vitro*. **(A)** Inhibition enzyme-linked immunosorbent assay (ELISA) was performed by growing mesangial cells in plates, followed by adding anti-dsDNA IgG that was premixed with d-form or scrambled ALW peptide. **(B)** The mRNA levels of SOCS1, Janus kinase 2 (JAK2), and signal transducer and activator of transcription 1 (STAT1) were determined in anti-dsDNA IgG-treated cells upon addition of d-form or scrambled ALW peptide. **(C,D)** Accordingly, the proteins of SOCS1, JAK2, and STAT1 were detected by Western blotting, followed by ImageJ quantitation of band intensities. In panels **(B–D)**, cells received 2-day treatment of anti-dsDNA IgG (2 µg/ml). Data were from three independent experiments. Data points and error bars represent mean ± SEM. Representative images are shown (*p < 0.05, **p < 0.01).

The effect of d-form ALW on the anti-dsDNA IgG regulation of SOCS1 signals was studied by culturing mesangial cells. It was found that d-form—but not scrambled ALW—inhibited anti-dsDNA IgG binding to mesangial cells (Figure 8A). Moreover, d-form ALW decreased the mRNA levels of JAK2 and STAT1 in anti-dsDNA IgG-treated cells (Figure 8B). Accordingly, the proteins of these molecules were determined by Western blotting and showed consistent changes in these cells upon addition of d-form ALW (Figures 8C,D). Furthermore, d-form ALW did not affect the mRNA expression level of SOCS1 but increased its protein level in these cells (Figures 8B,D). In addition, d-form ALW significantly reduced the mRNA expression levels of PDGFB, CTGF and Fn14 in mesangial cells stimulated by anti-dsDNA IgG (Figure S4 in Supplementary Material).

### Blocking Anti-dsDNA IgG Ameliorates SOCS1 Signals in SCID Mice

**Figure 9:**
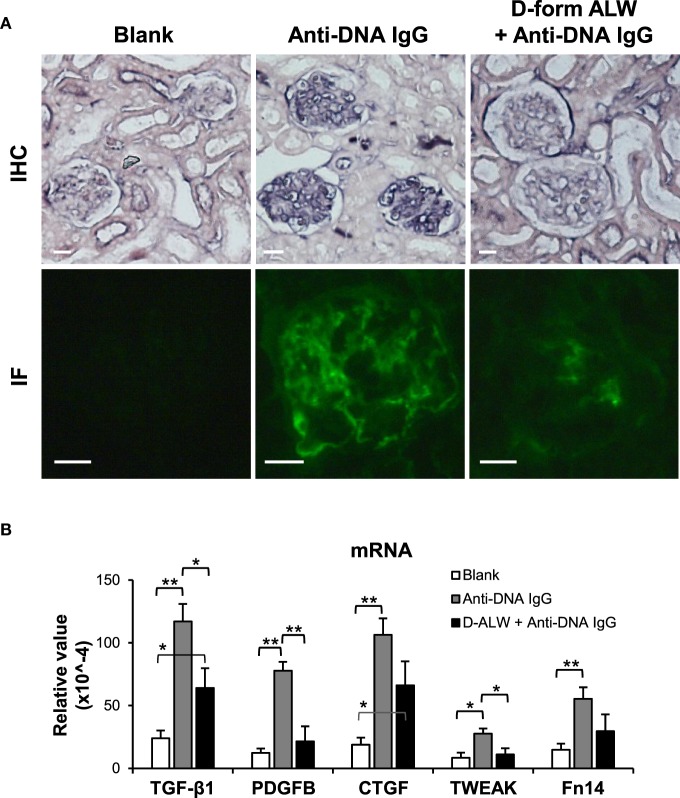
**The effect of d-form ALW peptide on renal IgG deposition and profibrotic factors in SCID mice**. Mice were intravenously injected with anti-double-stranded DNA IgG or plus d-form ALW peptide. **(A)** By immunohistochemistry (IHC) and immunofluorescence (IF), IgG deposition was detected in kidney tissues. **(B)** The mRNA expression levels of profibrotic cytokines [transforming growth factor (TGF)-β1, platelet-derived growth factor subunit B (PDGFB), and connective tissue growth factor (CTGF)] and proinflammatory factors (TWEAK and Fn14) were determined in fresh kidney tissues. There were five mice in each group. Data points and error bars represent mean ± SEM. Representative images are shown. Scale bar = 40 µm; *p < 0.05, **p < 0.01.

The effect of d-form ALW on anti-dsDNA IgG-injected SCID mice was studied by mixing anti-dsDNA IgG with ALW before such injection. Both IHC and IF demonstrated less IgG deposition in the glomeruli of these mice when compared with those treated with anti-dsDNA IgG alone (Figure 9A). Also, it showed substantial decrease in the mRNA levels of TGF-β1, PDGFB, and TWEAK after pre-mixture with d-form ALW (Figure 9B).

**Figure 10:**
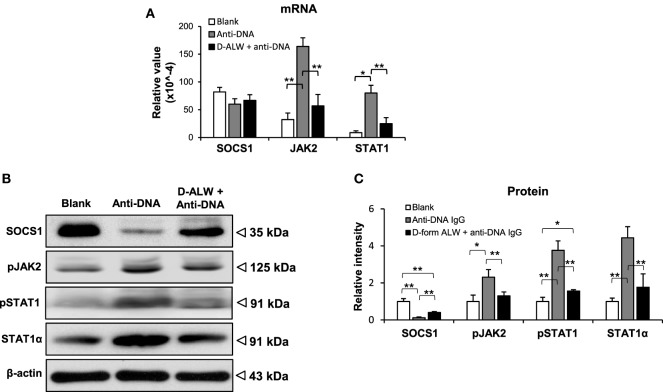
**The effect of d-form ALW peptide on anti-double-stranded DNA (dsDNA) IgG regulation of suppressor of cytokine signaling 1 (SOCS1) signals in SCID mice**. Mice were intrvenously injected with anti-dsDNA IgG or plus d-form ALW peptide. **(A)** The mRNA expression levels of SOCS1, Janus kinase 2 (JAK2), and signal transducer and activator of transcription 1 (STAT1) were determined in fresh kidney tissues. **(B,C)** By Western blotting, the proteins of SOCS1, JAK2, and STAT1 were detected kidney tissues accordingly. The band intensities were quantitated by ImageJ software. There were five mice in each group. Data points and error bars represent mean ± SEM. Representative images are shown (*p < 0.05, **p < 0.01).

The SOCS1 signals were evaluated in the kidneys of these mice. The mRNA levels of JAK2 and STAT1 were reduced in the d-form ALW plus anti-dsDNA IgG group when compared with anti-dsDNA IgG alone group, although the SOCS1 mRNA level was not affected (Figure 10A). Consistently, the expression levels of the SCOS1, JAK2, and STAT1 proteins showed remarkable attenuation in d-form ALW-treated group when compared with the anti-dsDNA IgG alone group (Figures 10B,C).

## Discussion

This study demonstrated that anti-dsDNA IgG-induced SOCS1 downregulation is pivotal in the pathogenesis of LN. The expression of SOCS1 decreases in kidney tissues of both patients with LN and MRL/lpr mice. Moreover, anti-dsDNA IgG blocks the SOCS1 signals in cultured kidney cells. Interestingly, anti-dsDNA IgG competes with JAK2 loop for SOCS1-KIR, and even catalyzes KIR directly. However, DNA-mimicking peptide abrogates such alteration of SOCS1 signals induced by anti-dsDNA IgG. Blockade of anti-dsDNA IgG ameliorates SOCS1 signals as well as profibrotic cytokines in SCID mice that are induced by anti-dsDNA IgG. Therefore, anti-dsDNA IgG suppresses SOCS1 signals and contributes to renal fibrosis.

Previous studies showed that anti-dsDNA IgG enhances the production of proinflammatory cytokines and fibrotic factors in cultured mesangial and tubular cells (12, 13). Consistently, we found that the profibrotic factors including TGF-β1, PDGFB, and CTGF have higher mRNA levels in kidneys of anti-dsDNA IgG-injected SCID mice. In fact, the SOCS1 mRNA level decreases significantly, whereas the expression of profibrotic genes increases in kidney biopsies from patients with LN (27). In this study, the SOCS1 expression decreases in kidneys of female MRL/lpr mice (24 weeks old) as well as anti-dsDNA IgG-injected SCID mice. It was reported that female MRL/lpr mice at earlier age (12 weeks old) exhibits an increase in SOCS1 mRNA level (37). This discrepancy might be due to a feedback response after the activation of the STAT1 pathway in LN at the early stage (37). Actually, significant increases in renal damage and proteinuria are observed in female MRL/lpr mice beginning when they are around 26 weeks old (33). Moreover, we found that anti-dsDNA IgG-injected SCID mice had elevated mRNA levels of TGF-β1, PDGFB, and CTGF in kidneys. In addition, the expression levels of TWEAK and Fn14 increase in kidneys of MRL/lpr mice. TWEAK and Fn14 activate in kidneys of mice with LN, contributing to glomerular and interstitial fibrosis by promoting the production of TGF-β1, monocyte chemotactic protein-1, interferon gamma-induced protein 10, and chemokine (C–C motif) ligand 5 (33, 38, 39). Therefore, the SOCS1 levels correlate negatively with anti-dsDNA IgG and profibrotic factors in LN.

A key phenomenon seen in our experiments is that anti-dsDNA IgG blocks the SOCS1 signals in kidney cells. The anti-dsDNA IgG-injected SCID mice had lower protein level of SOCS1 in their kidneys compared with other mice. Moreover, under anti-dsDNA IgG stimulation, mesangial cells express less SOCS1 but express more JAK2 and STAT1. Similar results were observed with other renal resident cells. Theoretically, higher SOCS1 level means lower JAK2 and STAT1 expression because the latter is inhibited under SOCS1 activation. Therefore, the decrease in SOCS1 but increase in downstream signals suggests suppression of SOCS1 signaling by anti-dsDNA IgG. However, the anti-dsDNA IgG treatment did not alter the mRNA expression levels of SOCS1 in both the kidneys of SCID mice and the cultured kidney cells, suggesting that anti-dsDNA IgG interferes with SOCS1 signaling at protein but not at mRNA level. On the other hand, the decrease in renal expression of SOCS1 mRNA was obvious in MRL/lpr mice, consistent with previous report that the kidneys of patients with LN express less SOCS1 mRNA (27). Such discrepancy in renal SOCS1 mRNA level between the anti-dsDNA IgG-injected SCID mice and the MRL/lpr mice as well as patients with LN may be because the latter have complexed deposition of other nephritogenic autoantibodies and also more severe inflammation in kidneys (8, 40).

Interestingly, anti-dsDNA IgG exhibits specific binding to SOCS1-KIR, which recognizes JAK2 loop and then suppresses JAK2/STAT1-mediated cytokine production. The KIR peptide not only has high affinity to JAK2 loop but also efficiently inhibits the binding of anti-dsDNA IgG to DNA antigen. This finding suggests a competition between KIR and DNA antigen in their interaction with anti-dsDNA IgG. Actually, DNA-mimicking peptides can cross-react with anti-dsDNA IgG and specifically block binding of anti-dsDNA IgG to multiple self-antigens (40–42). The algorithm of Basic Local Alignment Search Tool is used for comparing the sequences of KIR peptide and other proteins that are recognized by anti-dsDNA IgG (8, 11), showing that KIR peptide has high similarity with certain fragments of alpha actinin-4 (75%), laminin alpha 2 (83%), laminin gamma 2 (83%), and collagen type XXIV alpha 1 (67%) (Figure S9 in Supplementary Material). Collagen type XXIV alpha 1 is a component of matrigel, which also binds to anti-dsDNA IgG *in vitro* (8). So, KIR cross-reacts with anti-dsDNA IgG possibly by mimicking the antigenic property of DNA. Another important finding was that anti-dsDNA IgG catalyzes KIR peptide clearly. Although these results came from *in vitro* experiments, we can suppose that anti-dsDNA IgG harbors such catalytic activity intracellularly or even *in vivo*. In fact, anti-dsDNA IgG exhibits catalytic effects on bound self-antigens frequently, and the DNA cleavage and proteolysis are pivotal in the nephritogenicity of anti-dsDNA IgG (36, 43). Hence, the binding to and catalyzing of KIR of anti-dsDNA IgG may reasonably explain its inhibition of SOCS1 signaling, but without affecting the mRNA expression of SOCS1. The precise mechanism underlying KIR catalysis of anti-dsDNA IgG needs further study in future.

The DNA-mimicking peptides block the binding of anti-dsDNA IgG to self-antigens (40, 41). Since KIR peptide may initiate downstream JAK2/STAT1 signals *via* binding to JAK2 loop, ALW peptide was used alternatively for blocking anti-dsDNA IgG in our experiments. It was clear that ALW abrogates the inhibition of SOCS1 signals in kidney cells induced by anti-dsDNA IgG. Previous studies demonstrated that artificial peptides are susceptible to the catalyzing property of anti-dsDNA IgG (36, 44, 45). Moreover, d-form modification can endow peptides resistance to catalysis or improved stability without significantly affecting their biological activities (46, 47). d-Form peptides may even have improved oral bioavailability and enhanced binding activity and specificity with receptor or target proteins (48). In this study, d-form ALW not only inherits the inhibitory property of the l-form (natural) ALW, but also effectively abrogates inhibition of SOCS1 expression in kidney cells induced by anti-dsDNA IgG. The downstream JAK2/STAT1 signals are also attenuated accordingly. Therefore, d-form ALW peptide is valuable in ameliorating anti-dsDNA IgG-induced SOCS1 suppression in cells.

d-Form ALW peptide also ameliorates SOCS1 signals in anti-dsDNA IgG-treated SCID mice, which display glomerular IgG deposition and lupus-like renal damage (8). The IgG deposition achieves significant reduction as anti-dsDNA IgG being premixed with d-form ALW peptide. Accordingly, the elevated levels of profibrotic cytokines and proinflammatory factors decrease in kidneys. Since anti-dsDNA IgG induces renal damage through multiple mechanisms such as interfering with gene expression as well as metabolism and also enhancing cell proliferation (8), such amelioration of profibrotic cytokines may not be explained exclusively by restoring SOCS1 signals in kidney cells. However, SOCS1 expression decreases—whereas STAT1 level increases—in anti-dsDNA IgG-treated SCID mice, suggesting suppression of SOCS1 signals. The reduction of profibrotic cytokines in kidneys also reflects such suppressed SOCS1 signals. Hence, the *in vivo* study confirmed that anti-dsDNA IgG exerts fibrotic effect on kidneys *via* suppressing SOCS1 signals. Additionally, the pre-mixture of ALW peptide ameliorates such effect of anti-dsDNA IgG on SCID mice, further indicating DNA-mimicking peptides as potential prodrugs in the treatment of LN.

In conclusion, SOCS1 downregulation, JAK2/STAT1 activation, and production of profibrotic cytokines are prominent in kidneys with LN or anti-dsDNA IgG-stimulated renal cells. Anti-dsDNA IgG has high affinity to SOCS1-KIR and competes with JAK2 loop for binding sites in SOCS1-KIR, impeding the SOCS1 signals. The DNA-mimicking ALW peptide blocks anti-dsDNA IgG binding to SOCS1-KIR and consequently abrogates the phosphorylation of JAK2/STAT1α proteins as well as the elevation of profibrotic cytokines in kidney cells under anti-dsDNA IgG stimulation. Therefore, anti-dsDNA IgG participates in renal fibrosis of LN by suppressing SOCS1 signals, and DNA-mimicking peptide can restore such SOCS1 inhibitory effect and ameliorate renal fibrosis.

## Ethics Statement

This study was carried out in accordance with the recommendations of the guidelines of the Hospital Research Ethics Committee with written informed consent from all subjects. All subjects gave written informed consent in accordance with the Declaration of Helsinki. The protocol was approved by the Hospital Research Ethics Committee.

## Author Contributions

PW participated in the design of the study and performed most experimental work. JY, FT, ZD, and LX carried out some experiments. XL and KL discussed the experimental data and contributed to the interpretation of results. YX conceived and designed the study and prepared the manuscript. All the authors read and approved the final manuscript.

## Conflict of Interest Statement

The authors declare that the research was conducted in the absence of any commercial or financial relationships that could be construed as a potential conflict of interest.
