# Supplementary material for: Anti-Double-Stranded DNA IgG Participates in Renal Fibrosis through Suppressing the Suppressor of Cytokine Signaling 1 Signals
Source: Front Immunol. 2017 May 31;8:610. doi: 10.3389/fimmu.2017.00610 (PMC5449454; doi:10.3389/fimmu.2017.00610)
Supplement: Supplementary file 1 [file Data_Sheet_1.pdf]

Supplementary Table S1. Clinical information of kidney biopsies

No.	Sex (F/M)	Age (y)	Course (y/m)	Diagnosis	No.	Sex (F/M)	Age (y)	Diagnosis
1	F	27	2 y	LN	8[1]	M	56	Renal cell carcinoma
2	F	34	6 y	LN	9[1]	M	46	Renal cell carcinoma
3	F	41	5 m	LN	10[1]	F	39	Renal cell carcinoma
4	F	25	4 y	LN	11[1]	F	50	Renal cell carcinoma
5	F	42	6 m	LN	12[1]	M	21	Renal rupture
6	F	27	3 y	LN				
7	F	29	1 y	LN				

[1]Nonlesional kidney tissues were used as normal controls.

Abbreviations: F, female; M, male; y, year; m, month; LN, lupus nephritis

Supplementary Table S2. Sequences of primers

Oligonucleotide	Sequence
Collagen 1A1 primers	Forward: 5′-CCTAATGCTGCCTTTTCTGC-3′<br>Reverse: 5′-ATGTCCCAGCAGGATTTGAG-3′
CTGF primers	Forward: 5′-CCTGGTCCAGACCACAGAGT-3′<br>Reverse: 5′-GACAGGCTTGGCGATTTTAG-3′
Fibronectin 1 primers	Forward: 5′-AAGACCATACCTGCCGAATG-3′<br>Reverse: 5′-CAACTGGTTGGCATGAAATG-3′
Fn14 primers	Forward: 5′-CTAGTTTCCTGGTCTGGAGAAGATG-3′<br>Reverse: 5′-CCCTCTCCACCAGTCTCCTCTA-3′
IFN-γ primers	Forward: 5′-CACGGCACAGTCATTGAAAG-3′<br>Reverse: 5′-GCTGATGGCCTGATTGTCTT-3′
Nephrin primers	Forward: 5′-AGCGTAATCCTCTCCATCCT-3′<br>Reverse: 5′-ACACACAGGTGACCACATATTC-3′
PDGFB primers	Forward: 5′-CCCACAGTGGCTTTTCATTT-3′<br>Reverse: 5′-GTGGAGGAGCAGACTGAAGG-3′
Podocin primers	Forward: 5′-CGTCTCCAGACCTTGGAAATAC-3′<br>Reverse: 5′-CACATGGGCTAGACTGCTTAG-3′
TGF-β1 primers	Forward: 5′-TGCGCTTGCAGAGATTAAAA-3′<br>Reverse: 5′-GCTGAATCGAAAGCCCTGTA-3′
TWEAK primers	Forward: 5′-CGAGCTATTGCAGCCCATTAT-3′<br>Reverse: 5′-ACCTGCTTGTGCTCCATCCT-3′
GAPDH primers	Forward: 5′-CTCATGACCACAGTCCATGC-3′<br>Reverse: 5′-ACACATTGGGGGTAGGAACA-3′

Abbreviations: CTGF, connective tissue growth factor; IFN, interferon; PDGFB, platelet-derived growth factor subunit B; TGF, transforming growth factor; TWEAK, tumor necrosis factor-related weak inducer of apoptosis; GAPDH, glyceraldehyde 3-phosphate dehydrogenase

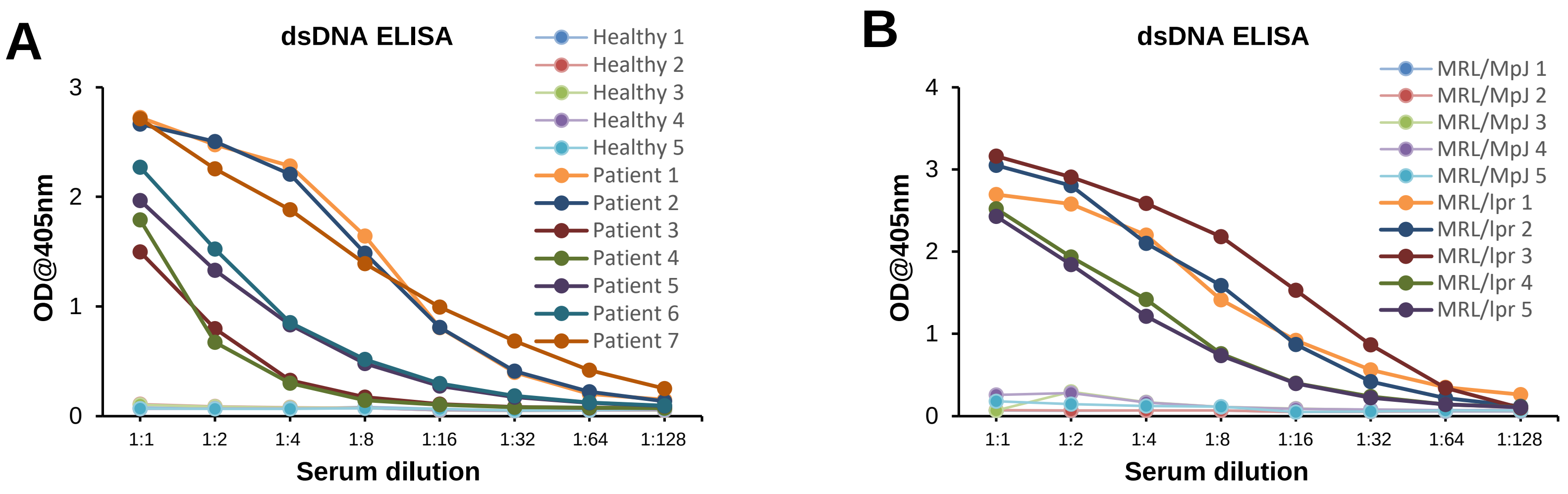

Supplementary Figure S1. Evaluation of serum anti-dsDNA IgG. The serum samples were collected from both human donors (normal controls and LN patients) and MRL/lpr (or MRL/MpJ) mice. **(A)** By ELISA, anti-dsDNA IgG was detected in sera from human donors. **(B)** Similarly, the serum anti-dsDNA IgG were detected in MRL/lpr and MRL/MpJ mice. The number of normal donors, patients with LN, MRL/lpr mice, and MRL/MpJ mice was 5, 7, 5, and 5, respectively. Representative images are shown.

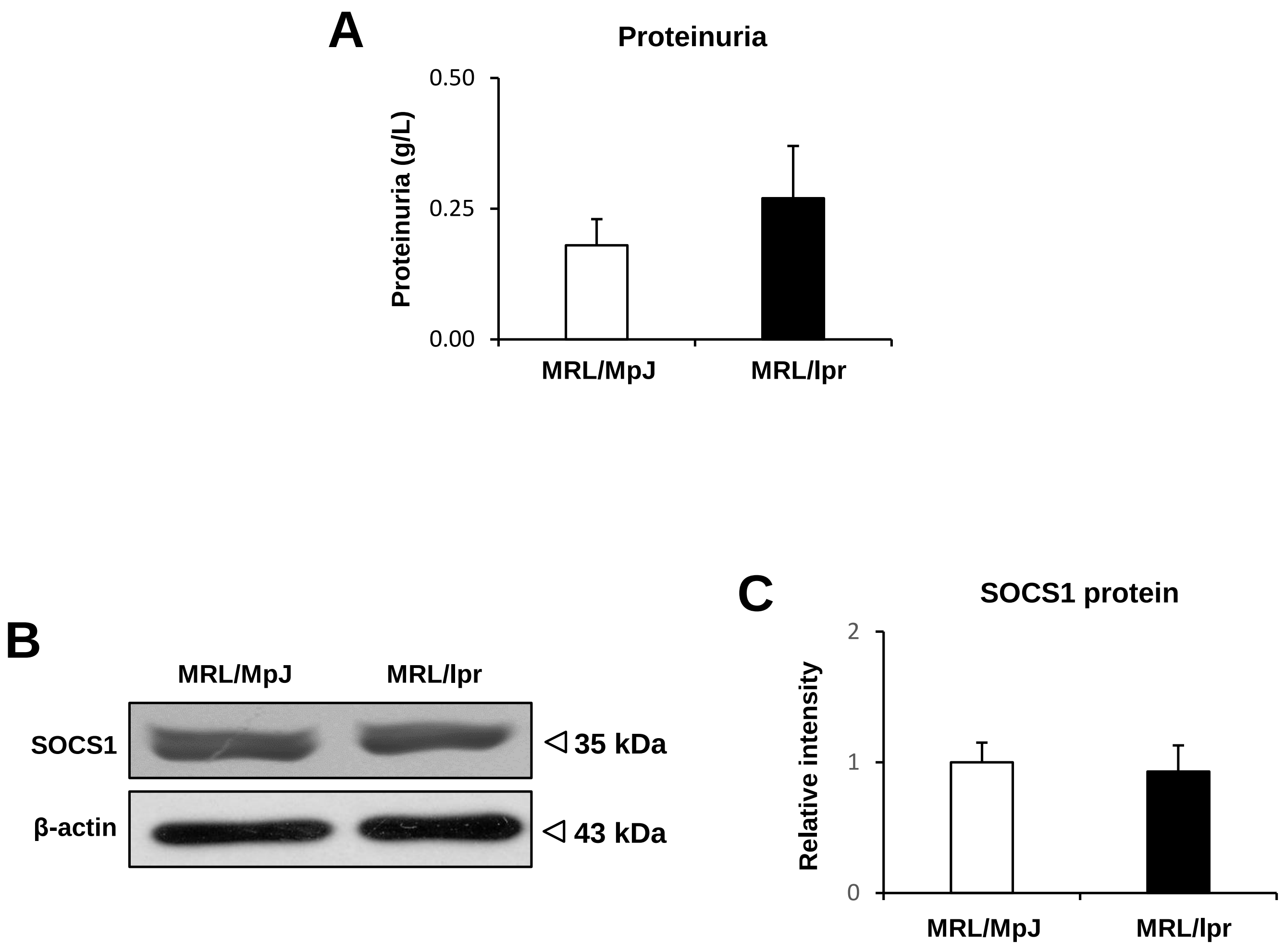

Supplementary Figure S2. Evaluation of proteinuria and renal levels of SOCS1 in MRL/lpr and MRL/MpJ mice (12-week old). (A) Proteinuria was determined in these mice. **(B)** By Western blotting, the protein expression levels of SOCS1 were determined. (**C**) The Western blot bands were quantitated by ImageJ software. There were 5 mice in each group. Representative images are shown.

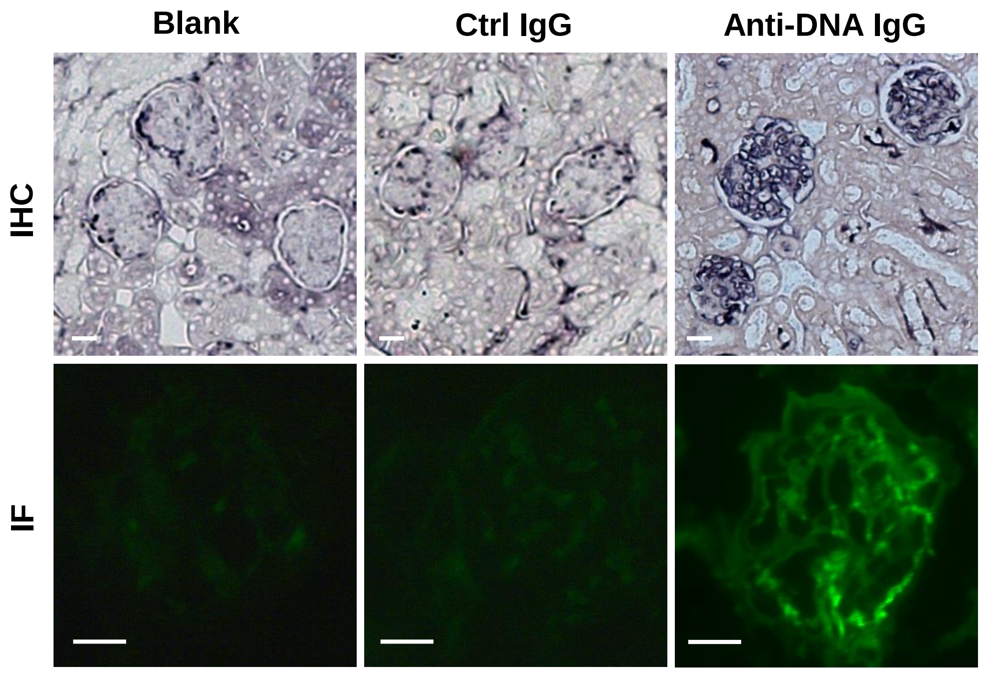

Supplementary Figure S3. **IgG deposition in kidneys of SCID mice.** Mice were intravenously injected with anti-dsDNA or control IgG before tissular analysis. By immunohistochemistry (IHC) and immunofluorescence (IF), IgG deposition was detected in glomeruli. There were 5 mice in each group. Representative images are shown. Scale bar = 40 µm.

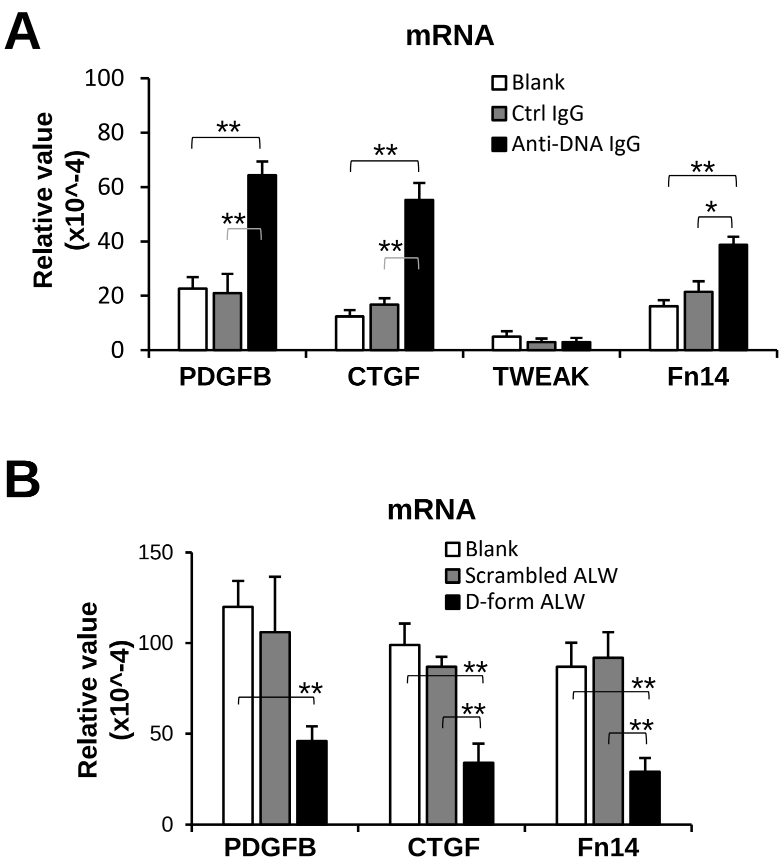

Supplementary Figure S4. T**he mRNA expression levels of proinflammatory factors in murine mesangial cells.** Cells were cultured in vitro and stimulated by control or anti-dsDNA IgG (2 µg/ml, 2 days). **(A)** The mRNA levels of PDGFB, CTGF, TWEAK and Fn14 were determined in these cells. **(B)** Cells were stimulated with anti-dsDNA IgG that was premixed with scrambled or D-form ALW peptide. Data were from three independent experiments. Data points and error bars represent mean ± SEM. *p < 0.05, **p < 0.01

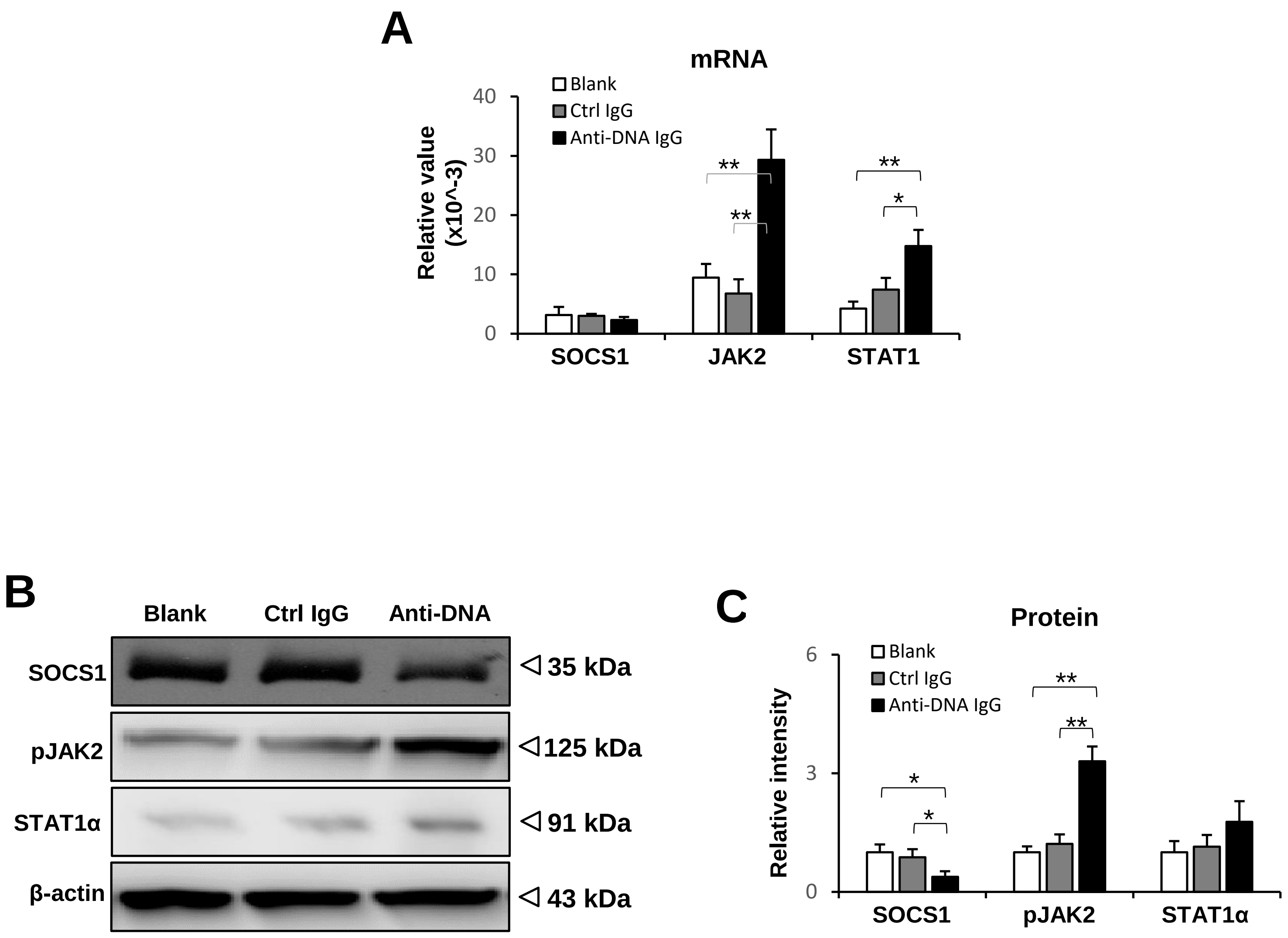

Supplementary Figure S5. **Evaluation of SOCS1 and JAK2/STAT1 expression levels in glomerular endothelial cells.** Cells were cultured in vitro and stimulated by control or anti-dsDNA IgG (2 µg/ml, 2 days). **(A)** The mRNA levels of SOCS1, JAK2, and STAT1 were determined in these cells. **(B)** Western blotting was performed to detect the proteins of SOCS1, JAK2, and STAT1 in cell lysates. **(C)** The intensities of the Western blot bands were quantitated by ImageJ software. There were no differences between the mice in blank and control IgG groups ($p > 0.05$). Data were from three independent experiments. Data points and error bars represent mean ± SEM. Representative images are shown. *$p < 0.05$, **$p < 0.01$

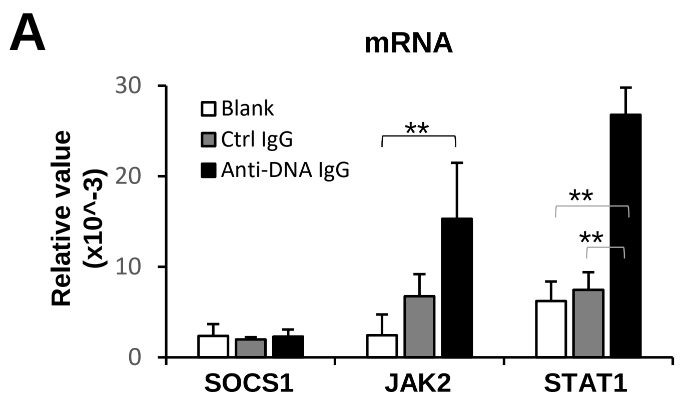

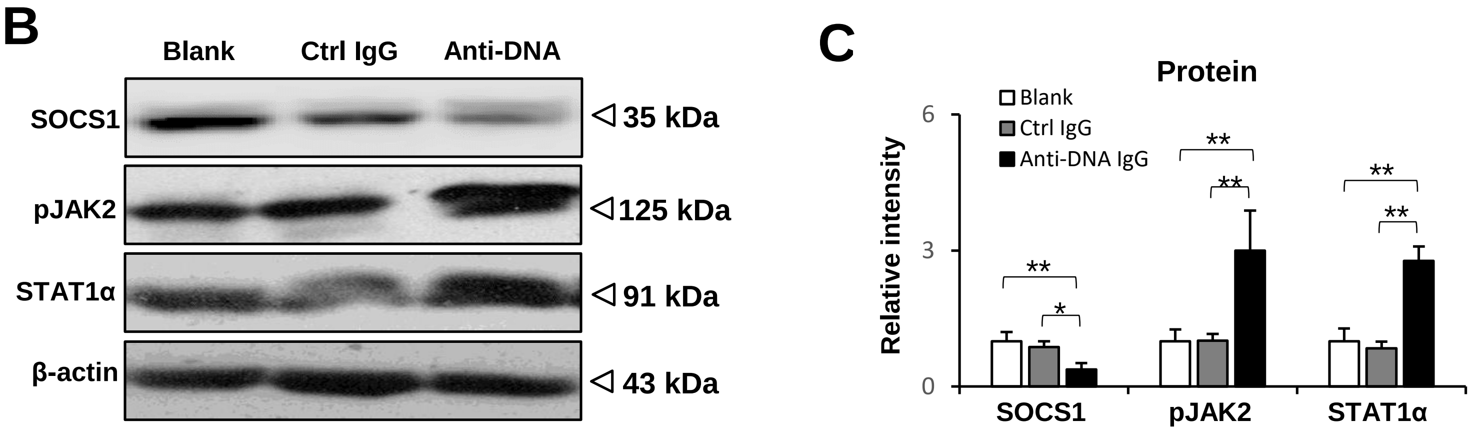

Supplementary Figure S6. **Evaluation of SOCS1 and JAK2/STAT1 expression levels in proximal tubular cells.** Cells were cultured in vitro and stimulated by control or anti-dsDNA IgG (2 μg/ml, 2 days). **(A)** The mRNA levels of SOCS1, JAK2, and STAT1 were determined in these cells. **(B)** Western blotting was performed to detect the proteins of SOCS1, JAK2, and STAT1 in cell lysates. **(C)** The intensities of the Western blot bands were quantitated by ImageJ software. There were no differences between the mice in blank and control IgG groups ($p > 0.05$). Data were from three independent experiments. Data points and error bars represent mean ± SEM. Representative images are shown. *$p < 0.05$, **$p < 0.01$

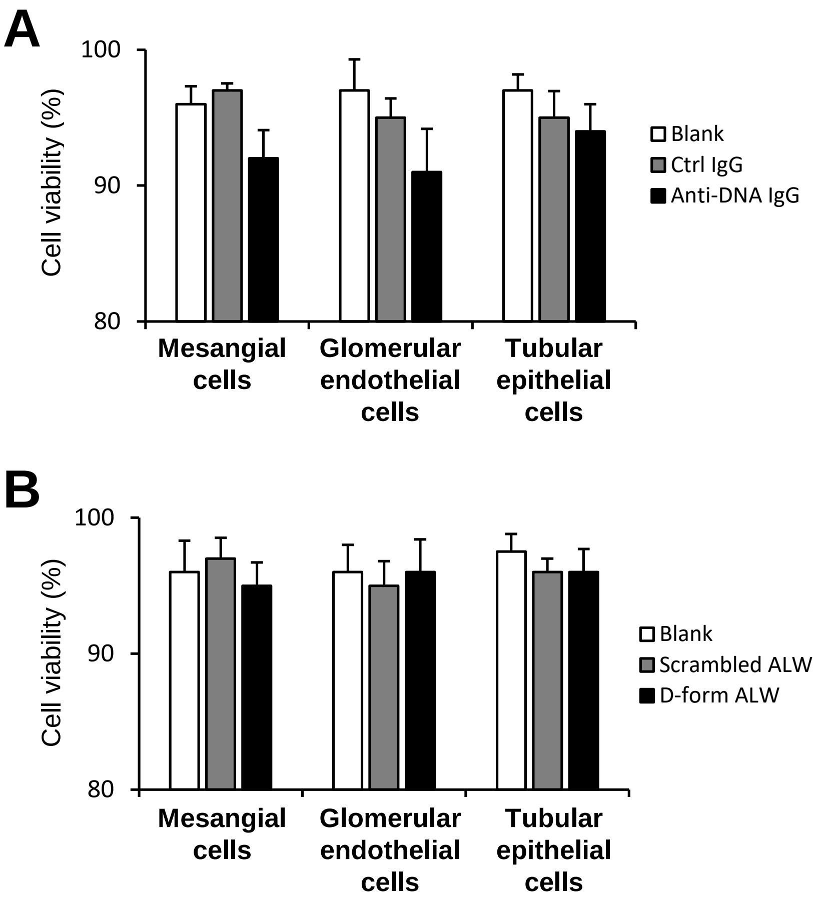

Supplementary Figure S7. **Cell viability assays of kidney cells. (A)** Murine cells were cultured in vitro, and received control or anti-dsDNA IgG stimulation (2 µg/ml, 2 days). **(B)** Similarly, these cells were also stimulated with scrambled or D-form ALW peptide (1 µg/ml, 2 days). The cell viability was measured using PrestoBlue viability reagent. Data were from three independent experiments. Data points and error bars represent mean ± SEM.

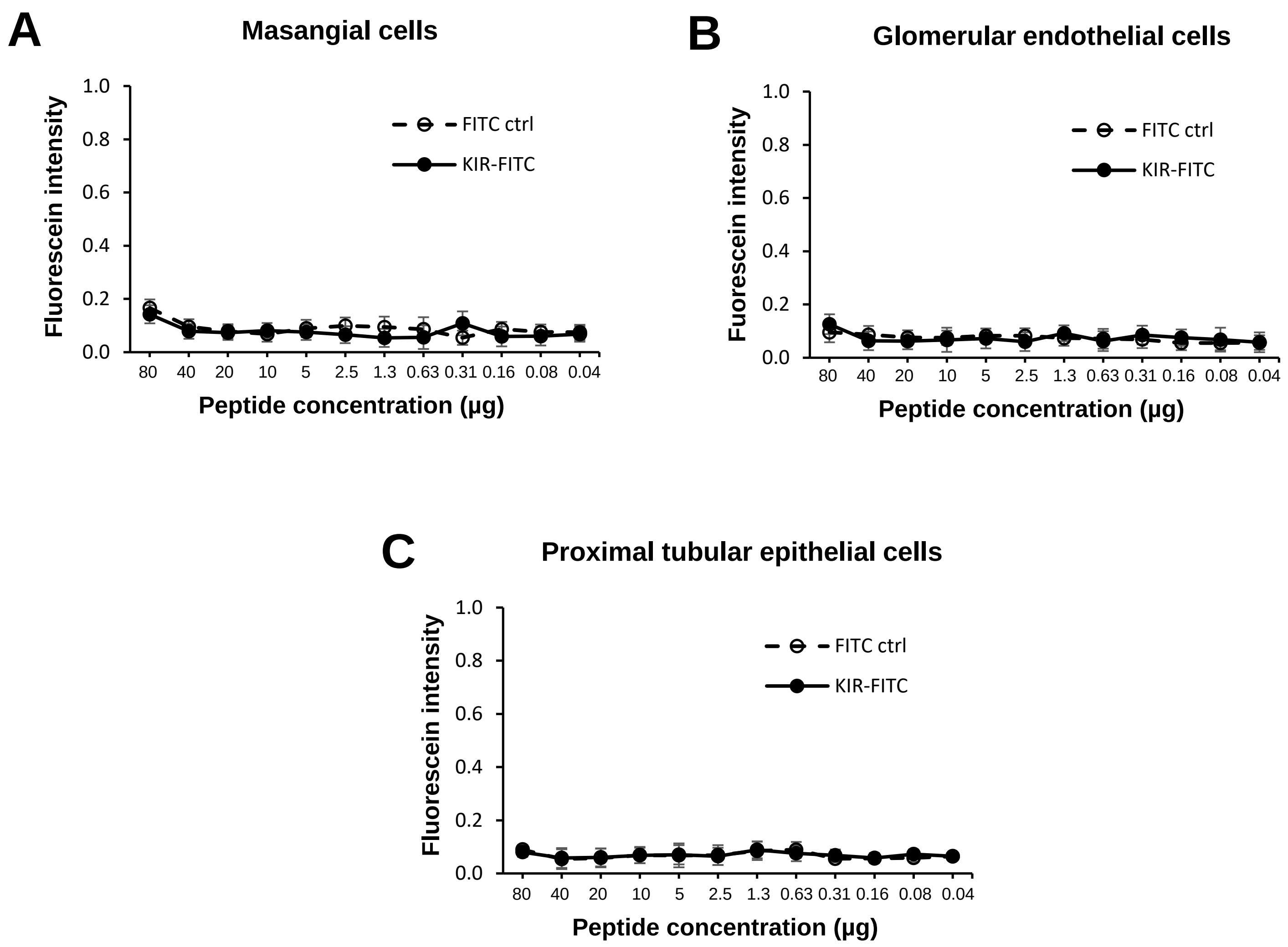

Supplementary Figure S8. **Cell surface ELISA for KIR binding to kidney cells.** Murine cells were cultured in vitro in 96-well plates and incubated with FITC-conjugated KIR or FITC alone (0-80 µg/ml). Plates were detected for fluorescein intensity under a Perkin Elmer Reader. **(A)** Mesangial cells were assayed for binding to KIR peptide. Similarly, glomerular endothelial cells **(B)** or proximal tubular epithelial cells **(C)** were assayed. Data were from three independent experiments. Data points and error bars represent mean ± SEM.

Mouse SOCS1：NP_001258532.1

KIR ↘

MVARNQVAADNAISPAAEPRRRSEPSSSSSSSSPAAPVRPRPCPAVPAPAPGDTHFRTFRSHSDYRRITRTSA
LLDACGFYWGPLSVHGAHERLRAEPVGTFLVRDSRQRNCFFALSVKMASGPTSIRVHFQAGRFHLDGSR
ETFDCLFELLEHYVAAPRRMLGAPLRQRRVRPLQELCRQRIVAAVGRENLARIPLNPVLRDYLSSFPFQI

Query 4 FRSHSDYRR 12 KIR
Sbjct 336 FR------DYRR 341 Alpha actinin-4 （75% similarity）

Query 1 FRTFRS 6 KIR
Sbjct 2364 FRTFSS 2369 Laminin, alpha 2 （83% similarity）

Query 3 TFR------------SH-----------S----DYRR 12 KIR
TFR SH S +YRR
Sbjct 316 TFRLNEHPSSHWSPQLSYFEYRR 338 Laminin, gamma 2 （83% similarity）

Query 3 TFRSHS 8 KIR
Sbjct 198 TFDPHS 203 Collagen, type XXIV, alpha 1 (67% similarity)

Supplementary Figure S9. **Similarities in amino acid residue sequence between KIR peptide and anti-dsDNA IgG-recognized proteins.** The algorithm of Basic Local Alignment Search Tool was used for the comparison of similarities of their sequences. The KIR sequence and certain fragments that have high similarity are indicated in red.
